# Expert considerations to guide the development and delivery of an exercise intervention in people with type 2 diabetes‐related foot ulcer disease

**DOI:** 10.1111/dme.70338

**Published:** 2026-04-30

**Authors:** Vicki L. Johnson, Vicky Hall, Rachel Berrington, Julia Burdon, Sally Schreder, Matthew McCarthy, Chris Brough, Patrick J. Highton, Michelle Hadjiconstantinou, Frances L. Game, Lisa J. Keogh, Solomon Tesfaye, Jonathan Valabhji, John R. Petrie, Ketan K. Dhatariya, Tom Yates, Melanie J. Davies, Kamlesh Khunti

**Affiliations:** ^1^ Leicester Diabetes Centre University Hospitals of Leicester NHS Trust Leicester UK; ^2^ Diabetes Research Centre, College of Life Sciences University of Leicester Leicester UK; ^3^ National Institute for Health and Care Research Applied Research Collaboration East Midlands Leicester UK; ^4^ NIHR Biomedical Research Centre University of Leicester Leicester UK; ^5^ Department of R&D University Hospitals Derby and Burton NHS Foundation Trust Derby UK; ^6^ Sheffield Teaching Hospitals NHS Foundation Trust Sheffield UK; ^7^ Department of Metabolism, Digestion and Reproduction, Faculty of Medicine Chelsea and Westminster Hospital Campus, Imperial College London London UK; ^8^ Robertson Centre for Biostatistics/ Glasgow Clinical Trials Unit, School of Health & Wellbeing, College of Medical, Veterinary and Life Sciences University of Glasgow Glasgow UK; ^9^ Elsie Bertram Diabetes Centre Norfolk and Norwich University Hospitals NHS Foundation Trust Norwich UK; ^10^ Norwich Medical School University of East Anglia Norwich UK

**Keywords:** diabetes mellitus, type 2 foot ulcer exercise

## Abstract

**Objective:**

Engaging in exercise presents unique challenges for those with type 2 diabetes (T2D) and diabetes‐related foot ulcer disease (DFUD). This brief report describes the development of practical considerations aiming to support exercise in people with T2D and DFUD.

**Research Design and Methods:**

An expert group of multidisciplinary healthcare professionals (HCPs), researchers and individuals with DFUD reviewed available evidence and developed practical considerations for the assessment, prescription and monitoring of exercise within an ongoing randomised controlled trial.

**Results:**

Key considerations include screening for contraindications, individualising exercise prescription, determining weight‐bearing status and initial supervision by HCPs. Self monitoring of exercise and appropriate foot protection advice and care are also encouraged.

**Conclusions:**

We have developed practical considerations to use when delivering an exercise intervention within a study aiming to improve cardiovascular health for people with T2D and DFUD. For this specific population, these recommendations include prescribing non‐weight bearing physical activity (such as arm ergometry or chair‐based upper limb exercises) for those with active foot ulcers, educating and encouraging footcare self‐management (including how to check feet and when to seek advice), monitoring glucose levels in participants at high risk of hypoglycaemia or hyperglycaemia and reducing the barriers to exercise. Pragmatic measures aimed at maintaining safety include determining the level of pre‐assessment and individualised monitoring based on clinical presentation and/or risk as well as equipment and staff resource. This approach may also be helpful when supporting exercise in a wider population.

## Graphical abstract

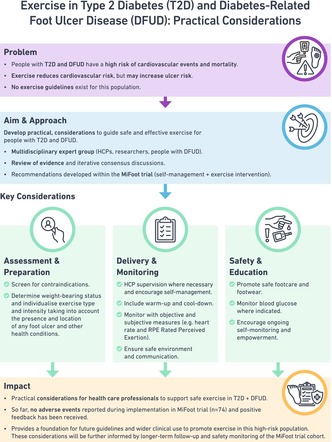

**What's new?**

**What is already known?**

- People with type 2 diabetes‐related foot ulcer disease (T2DFUD) have a high risk of cardiovascular disease events, re‐ulceration and mortality. Exercise can help reduce cardiovascular risks in the long‐term, but exercise poses potential risks of re‐ulceration. There are currently no exercise guidelines specifically for this group of people.

**What did we find?**

- We developed practical considerations to support exercise for people with T2DFUD as part of a complex intervention ‘MiFoot’.

**What are the implications?**

- These considerations are being used within an ongoing trial which may also be useful for healthcare professionals to provide pragmatic exercise advice for people with DFUD.

## 1 INTRODUCTION

Physical activity (including daily activity as well as structured exercise) is recommended both for people with Type 2 Diabetes (T2D)^1^ and cardiovascular disease (CVD).^2^ However, for those with Diabetes‐related Foot Ulcer Disease (DFUD), who have a high risk of ulcer exacerbation/re‐ulceration and delayed healing, CVD events^3^ and mortality (especially if ischaemia is present),^4^, ^5^ engaging in exercise presents unique challenges. In the absence of formal guidelines, we report the development of considerations by a multidisciplinary group for use during a trial that supports exercise for this population.

## 2 METHODS

A multidisciplinary group of 14 UK healthcare professionals (HCPs) and researchers (including physiotherapists, diabetes and advanced specialist nurses, cardiac nurses, podiatrists, cardiac rehabilitation specialists, exercise professionals, consultants in T2D and DFUD, clinical psychologist and a general practitioner) along with individuals living with DFUD, held formal meetings and discussions over a 2‐year period during the development of an intervention called ‘MiFoot’. MiFoot consists of self management education and exercise sessions, currently being tested in a randomised controlled trial.^6^ The expert group involved in MiFoot, iteratively developed, reviewed and refined considerations to support self management and exercise in people with DFUD.

### 2.1 Evidence

There are well established benefits and recommendations for physical activity and exercise, which often outweigh risks. Physical activity can be defined as any bodily movement that requires energy expenditure. This can include walking, active transport, hobbies and activities of daily living (e.g. shopping, cooking and getting dressed) and is usually carried out independently. Exercise is a subset of physical activity and may be defined as such if it is planned, structured, repetitive and/or being carried out to benefit health. People may exercise independently or take part in exercise programmes or be referred to services to support or supervise exercise, such as cardiac rehabilitation. Adults are recommended to be active daily, with a goal of 150 min of moderate or 75 min of vigorous activity per week, plus strength and balance exercises twice weekly, and to break up prolonged sitting.^7^, ^8^ Adopting these healthy physical behaviours supports T2D management, reduces CVD risk and improves overall well‐being.^1^, ^9^

The potential risks of physical activity include falls, cardiac events, glycaemic episodes or delayed healing of an ulcer or re‐ulceration. People with active foot ulcers are advised to avoid weight‐bearing activity, if the ulcer is on a weight‐bearing surface, to allow ulcerated tissue to heal, thus reducing engagement in traditional structured exercise.^10^, ^11^, ^12^ Moreover, peripheral neuropathy, off‐loading devices, falls risk, neuropathic pain, peripheral arterial disease, co‐morbidities and lack of knowledge or confidence in appropriate exercise as well as physical, psychological and social barriers can significantly reduce activity levels,^13^, ^14^ further increasing CVD risk and the risk of poor health. Therefore, there is a need for tailored interventions that promote and support exercise while adhering to footcare guidelines^15^ so as not to exacerbate CVD or foot ulcer risk.

Screening and assessment prior to commencing (or increasing) exercise may be necessary or considered standard practice, to assess or manage risk, especially within healthcare settings. Various options exist: from no medical clearance needed (e.g. if a person is already physically active, takes part in exercise and/or has no medical conditions); to using clinical judgement (e.g. if someone has a medical condition but is already highly physically active or if someone may have symptoms of a new undiagnosed medical condition); and/or exercise testing (e.g. a walking test may be standard practice within cardiac rehabilitation assessments or to investigate cardiac symptoms). Of note, exercise tests have not been shown to influence CVD outcomes in asymptomatic individuals and therefore may not always be necessary.^1^, ^9^, ^16^, ^17^, ^18^

## 3 RESULTS

Our considerations for the trial intervention focussed on pragmatic assessment, prescription and monitoring of exercise, whilst promoting empowerment, health and well‐being and reducing barriers to physical activity. These are summarised in Table 1 and described below.

**TABLE 1:** Practical considerations.

1	Participants should be screened and assessed for contraindications prior to commencing exercise
2	Provide advice regarding the type and intensity of exercise
3	Presence and location of an active foot ulcer should be considered to determine weight bearing status for exercise
4	Educate and encourage footcare
5	Reduce barriers to exercise, while maintaining safety
6	Conduct an adequate warm up and cool down
7	Appropriately and pragmatically monitor response to exercise
8	Educate and encourage self‐monitoring
9	Appropriately supervise exercise
10	Monitor glucose levels in participants at high risk of hypoglycaemia or hyperglycaemia
11	Ensure appropriate communication
12	Exercise in a safe environment

### 3.1 Participants should be assessed for contraindications prior to commencing exercise

Participants should be screened for diagnosed and potentially undiagnosed contraindications^18^ (such as unstable angina or acute heart failure) before starting exercise, to reduce the risk of exercise‐related cardiac‐events, especially given the high prevalence of cardiac autonomic neuropathy in this population.^19^ This can be achieved by reviewing medical history and current symptoms (Appendix S1, page 3). Assessment via a resting 12‐lead electrocardiogram^20^ and vital signs^19^ (Appendix S1, page 2) may also be considered, especially if promoting higher intense exercise, to assess for potential undiagnosed contraindications. If contraindications are present or suspected, referral for further assessment and/or management is recommended prior to exercise (e.g. via cardiac services).

### 3.2 Provide advice regarding the type and intensity of exercise

Exercise advice should be based on assessment findings,^21^ considering the severity of neuropathy, CVD, musculoskeletal, balance or mobility issues, presence and location of any active foot ulcer (and associated management) and current physical activity levels^22^ to reduce falls or CVD risk. Exercise recommendations should include intensity (none, light, moderate or vigorous) and type of exercise (e.g. strengthening, balance, chair‐based).^23^ Light exercise or activity (e.g. activities of daily living) is not usually contraindicated and can be encouraged. Vigorous exercise is generally not suitable for deconditioned individuals or those who do not regularly exercise due to the risk of precipitating CVD events or musculoskeletal injury. For MiFoot, light exercise is initially prescribed for those with established CVD or who do not regularly undertake physical activity, and moderate exercise for those with no CVD restrictions and/or who already undertake regular physical activity.

### 3.3 Presence and location of an active foot ulcer should be considered to determine weight bearing status for exercise

Exercise (especially weight‐bearing e.g. brisk walking) could increase the risk of re‐ulceration. However, exercise can come in various forms, including upper‐body and chair‐based exercises, which can improve hyperglycaemia^24^ and circulation, reduce friction and may delay the progression of peripheral neuropathy,^22^, ^25^ thereby potentially benefiting the underlying ulcer pathophysiology. Non‐weight bearing exercise should be advised (in preference to weight‐bearing exercise) if an active foot ulcer is present on a weight‐bearing surface, to reduce the risk of delayed healing or ulcer exacerbation. Furthermore, if performing an exercise test, consider the foot ulcer status to determine the type of exercise test. For example, if an active foot ulcer is present, a non‐weight bearing exercise test such as an arm ergometry protocol should be followed instead of a walking test to reduce the risk to the feet.

### 3.4 Educate and encourage footcare

Care should be taken when being active to minimise any risk to feet by wearing appropriate footwear (e.g. offloading devices)^15^ and checking feet before and after exercise.^13^, ^22^ Footcare advice is provided during the MiFoot intervention through explanations and videos, reminders before and after each exercise session and individual support where needed. Utilising existing resources, for example footcare advice leaflets, services or websites such as Diabetes UK https://www.diabetes.org.uk/about‐diabetes/looking‐after‐diabetes/complications/feet#prevent can be helpful.

### 3.5 Reduce the barriers to exercise, while maintaining safety

Undertaking assessments such as exercise tests (e.g. shuttle walk test or exercise electrocardiogram) may be useful for some participants and can be useful to prescribe exercise. However, they also require significant resources (staff, space and equipment), which are not always necessary^9^ or implementable. Individuals should be encouraged to safely engage in independent daily physical activity^7^, ^8^ to promote ownership and control over their own health and not be solely reliant on supervised exercise from HCPs (e.g. cardiac rehabilitation) or devices (e.g. heart rate monitor), due to limited access. Appropriate screening and assessment prior to exercise (also see consideration 1), monitoring according to need during exercise (see considerations 7 and 8) and optimal medical management of an individuals' diabetes and CVD risk factors can help maintain safety.

### 3.6 Conduct an adequate warm up and cool down

Gradual warming up before and cooling down after exercise^23^ reduces the risk of musculoskeletal injury and CVD events.^18^ Demonstration and instruction of a warm‐up and cool‐down (e.g. by an HCP) can then be replicated, facilitating independent activity.

### 3.7 Appropriately and pragmatically monitor response to exercise

Response to exercise may be assessed and monitored via an exercise assessment, electrocardiogram (ECG), heart rate monitoring and/or subjectively, either through self monitoring or monitoring from a HCP. In the context of MiFoot, monitoring heart rate (HR) during the first three sessions only (unless clinically indicated to continue), alongside the Borg Rated Perceived Exertion (RPE) scale,^26^ was deemed a reasonable and pragmatic way to monitor participants safely^27^ and adjust exercise (e.g. intensity) where necessary, while minimising barriers (also see considerations 3, 5 and 9). HR can be monitored in several ways (e.g. ECG, chest or wrist monitors), with some methods being easier to implement in practice than others. HR targets can be calculated using the Karvonen method.^22^, ^27^ In the T2D and CVD literature, moderate activity varied between 40 and 70% of HR reserve (HRR) and RPE between 11 and 13^16^, ^23^, ^28^ with a lower RPE recommended for those with lower levels of physical activity. Taking into account potential autonomic neuropathy and blunted heart rate response,^22^ we agreed that light exercise would be deemed <40% HRR / RPE 9–11 and moderate exercise <60% HRR / 12–13 RPE.^23^ Participants should not exceed their prescribed level. The HR monitoring device should be carefully considered, ensuring reliability as well as practicality. In the context of MiFoot, a wrist‐worn HR monitor was deemed practical (easy to fit on multiple participants within a group setting), with the choice of device being determined by usability (patient preference, having a large enough wrist strap for those who may be living with overweight and large display in case of retinopathy).

### 3.8 Educate and encourage self monitoring

Education should include advising individuals to monitor for any potential changes in foot condition as well as exertion, and to understand their prescribed level of exercise and potential adverse symptoms (e.g. chest pain) to reduce the risk to feet and CVD risk. Combining subjective (RPE) and objective (HR) exertion measures supports safe exercise, encourages unsupervised activity (e.g. at home) and builds self management and confidence. This is particularly important for individuals who may experience autonomic neuropathy, altered perception of exertion or a blunted physiological response to exercise.^22^ By checking their feet before and after exercise, if any redness or changes are noted, individuals should be aware and encouraged to seek HCP advice (e.g. via their doctor, nurse or local foot specialist service).

### 3.9 Appropriately supervise exercise

For motivated, confident, low‐risk individuals, supervision of exercise may not be necessary. However, HCP supervision facilitates adherence to the prescribed level, monitoring and safe progression or adjustment of exercise. For example, HCPs can adjust exercise intensity depending on any changes in health status (for example decrease intensity if an individual is sick; increase if a health condition such as asthma becomes better managed) and response to exercise (e.g. decrease intensity if an individual is finding it too hard or HR increases above the prescribed target), and also adapt the type of exercise depending on any foot ulcer presence (e.g. advise standing exercise if a foot ulcer heals). Supervision should continue for 15 min post‐exercise to monitor for potential adverse events (e.g. hypoglycaemia or post‐exercise syncope).

### 3.10 Monitor glucose levels for participants at risk of hypoglycaemia or hyperglycaemia

Glucose levels can be measured by finger‐prick testing or, for those who have access, via continuous glucose monitoring (CGM). Glucose levels should be checked before, during and after exercise for those at high risk, particularly individuals taking medications that increase the risk of hypoglycaemia (e.g. insulin and sulfonylureas). If glucose is <4.0 mmol/L (72 mg/dL), then hypoglycaemia should be treated effectively before exercise (e.g. through ingestion of fast‐acting carbohydrate). Individuals with a high HbA1c or where their medication regime is not optimised, may present with hyperglycaemia. If glucose levels exceed 17.0 mmol/L (304 mg/dL), then exercise may be limited to light intensity. Exercise would be contraindicated if associated with symptoms that may indicate diabetes emergencies such as ketoacidosis or hyperosmolar hyperglycaemic state,^29^ in which case emergency care must be urgently sought. Reviewing an individual's diabetes medication regime may be necessary, as well as education about how their medication works, how to take it and potential alternative options, to optimise diabetes management and reduce the risk of hypo‐ or hyperglycaemia.

### 3.11 Ensure appropriate communication

Clear written and verbal communication is essential between HCPs assessing and supervising participants.^18^, ^23^ A bespoke assessment form (Appendix S1) and monitoring sheet (Appendix S2) were developed for MiFoot. These can be used to systematically assess and record appropriate information to prescribe, monitor, encourage and progress exercise.

### 3.12 Exercise in a safe environment

The venue should be an appropriate size, easily accessible, adequately ventilated, with emergency equipment available (e.g. automated external defibrillator). Hydration should be encouraged throughout to prevent post‐exercise complications. Equipment should be appropriate for all (including chairs with arms and bariatric seating), maintained and participants instructed prior to use where necessary. Staff should receive adequate training (e.g. basic life support, identifying and managing deteriorating patients) and be aware of emergency procedures.

Documentation to assist with these considerations can be found in Appendices S1 and S2. These considerations have been applied to the delivery of the MiFoot intervention with 74 participants to date, and no adverse events have been reported in relation to exercise safety. Feedback from participants has also been positive so far.

## 4 DISCUSSION

To our knowledge, these are the first set of practical considerations to support exercising people with T2D and DFUD. In the absence of formal guidelines, we used the available evidence and expert input, including from individuals with lived experience, to develop considerations that have formed the basis of a tailored exercise intervention currently being tested within a clinical trial.

Limitations include that no formal systematic review or Delphi consensus was undertaken. The study also remains ongoing, with adverse event reporting and outcome measures being collected at 12 and 24 months. This may lead to the future confirmation or refinement of our considerations.

We have developed these considerations to guide HCPs when delivering exercise to people with T2D and DFUD within the context of an ongoing clinical trial. In the absence of any other formal guidelines, these considerations may also be useful for HCPs to consider when supporting physical activity in this high‐risk population.

## AUTHOR CONTRIBUTIONS

VJ, VH, JB and RB prepared the manuscript; KK developed the idea. VJ, VH, RB, JB, SS, MM, CB, FG, LK, ST, JV, MD and KK were part of the expert panel. PH, MH, VJ, VH, RB, JB, SS, MM, CB, FG, LK, ST, JV, MD, KK supported the development and execution of the MiFoot intervention and study. JP, KD and TY provided further expertise and review of the recommendations. All authors reviewed and approved this manuscript. KK acts as guarantor.

## FUNDING INFORMATION

MiFoot is funded by the National Institute for Health and Care Research (NIHR) under its Programme Grants for Applied Research Programme and Diabetes UK (NIHR202021). The views expressed in this publication are those of the author(s) and not necessarily those of the NIHR, NHS or the Department of Health and Social Care.

## CONFLICT OF INTEREST STATEMENT

KK has acted as a consultant, speaker or received grants for investigator‐initiated studies from Abbott, Astra Zeneca, Bayer, Hikma, Novo Nordisk, Sanofi‐Aventis, Servier, Lilly and Merck Sharp & Dohme, Boehringer Ingelheim, Oramed Pharmaceuticals, Pfizer, Roche, Daiichi‐Sankyo, Applied Therapeutics, Embecta and Nestle Health Science. MJD has acted as a consultant/advisor and speaker for Eli Lilly, Novo Nordisk and Sanofi, has attended advisory boards for AbbVie, Amgen, AstraZeneca, Biomea Fusion, Carmot/Roche, Daewoong Pharmaceutial, Sanofi, Zealand Pharma, Regeneron, GSK and EktaH and as a speaker for AstraZeneca, Boehringer Ingelheim and Zuellig Pharma. She has received grants from AstraZeneca, Boehringer Ingelheim and Novo Nordisk. PH has received honoraria from Servier. JV is the National Specialty Advisor for Multiple Long‐Term Conditions at NHS England, and was the National Clinical Director for Diabetes and Obesity at NHS England from April 2013 to September 2023. JRP reports personal fees (via his employing institution) from Merck KGaA (Lectures), research support from Merck KGaA (Grant), personal fees from IQVIA (Boehringer Ingelheim Adjudication Committees) and travel support from Sanofi. Dr. Petrie has received non‐financial support as co‐CI of a JDRF/Breakthrough T1D‐funded trial (NCT03899402) from Astra Zeneca (donation of investigational medicinal product to US site only) and Novo Nordisk [donation of investigational medicinal product to UK site only; supplementary financial support (to mitigate a budget cut during the COVID‐19 pandemic)]. KKD has received honoraria, travel or attended advisory boards from Abbott Diabetes, AstraZeneca, Boehringer‐Ingelheim, Eli Lilly, Menarini, Novo Nordisk, Roche and Sanofi Diabetes. VJ, SS, JB, VH and CB are employed through the University Hospitals of Leicester NHS Trust, which holds the Intellectual Property rights for and receives not‐for‐profit income for the DESMOND suite of programmes (which includes MiFoot). ST reports honoraria for educational meetings from Viatris, Worwag Pharma, Novo Nordisk, Merk, AstraZeneca, Nevro, P&G Health, Viatris, Medtronic and Grunenthal; consulting fees from Worwag Pharma, Grunenthal, Merz, unrestricted research grants from Viatris, Proctor and Gamble and Withings. RB, MM, MH, LT, FG, TY have no conflicts of interest to declare.

## Supporting information


**Appendix S1.** MiFoot assessment form.

**Appendix S2.** MiFoot physical activity monitoring sheet.

## Data Availability

Data sharing not applicable to this article as no datasets were generated or analysed during the current study.
