# Supplementary material for: Expert considerations to guide the development and delivery of an exercise intervention in people with type 2 diabetes‐related foot ulcer disease
Source: Diabet Med. 2026 Apr 30;43(8):e70338. doi: 10.1111/dme.70338 (PMC13380388; doi:10.1111/dme.70338)
Supplement: Supplementary file 1 — Appendix S1. MiFoot assessment form. [file DME-43-e70338-s001.pdf]

Patient NHS No:

Addressograph:
Name:
Address:
NHS No:
DOB:

MiFoot

Leicester Diabetes Centre

MIFOOT ASSESSMENT FORM

BASELINE RESEARCH APPOINTMENT

Study ID MiF -			-			

Telephone home:		Mobile:	
NOK contact details<br><br>(inc. relationship)		Email:<br><br>Own/family/friend/ Doesn't have one	
Requires Transport	Yes ☐ No ☐		
Social Circumstances	Lives alone ☐ Lives with spouse ☐<br>Lives with family ☐ Other ☐<br>Details:		
Occupation / Employment status			
GP details			
Current respect form in place? (check notes only, not for discussion)		If yes, attach copy to this form	

Staff name:	Designation:
Date:	Signature:

Patient NHS No:

OBSERVATIONS

Date bloods taken:<br>(From GP records)		Resting Heart Rate	
Weight (kg)		Resting SpO2 (%)	
Height (cm)			
BMI<br>nhs.uk/live-well/healthy-weight/bmi-calculator/		Sitting BP<br>(Average of 3 readings)	
Waist circumference (cm)		Standing BP	
Waist : Height Ratio<br>Waist cm / height cm		Total Cholesterol	
HbA1c (mmol/mol)		LDL Cholesterol	
Random Glucose		eGFR	
Liver Function Tests		ACR	
Notes			

Staff name:	Designation:
Date:	Signature:

Patient NHS No:

EXERCISE SAFETY (ACSM CONTRAINDICATIONS)

**Does the participant have any of these contraindications to exercise?**	**Y/N**	**Further details**
Unstable or acute heart failure		
Unstable angina or diagnosis within last month		
Uncontrolled arrhythmias		
Tachycardia (> 100bpm)		
Febrile illness		
Systolic BP >180 and/or Diastolic BP >100		
Symptomatic hypotension		
AAA >5.5cm		

**Symptoms**	**Y/N**	**Further details**
Chest discomfort with exertion		
Unreasonable breathlessness		
Dizziness, fainting, blackouts		
Ankle swelling		
Unpleasant awareness of forceful, rapid or irregular heart rate		
Burning/cramping in lower limbs when walking short distances		

Staff name:	Designation:
Date:	Signature:

Patient NHS No:

Has the participant had, or do they currently have:	Y/N		Further details
Heart attack			
Heart surgery, cardiac catheterisation, or coronary angioplasty			
Pacemaker/implanted cardiac defibrillator/rhythm disturbance			
Heart valve disease			
Heart transplant			
Congenital heart disease			
Diabetes (circle)	T1	T2	
Renal disease			

Any other Current Medical Condition(s)

- (Inc. any new symptoms, recent changes to existing symptoms, or conditions that may impact on ability exercise)

Complete in conjunction with above

Any other Past Medical History

- (Inc. vascular, metabolic, musculoskeletal, cardiac, neurological, infectious diseases, GI, Ca, mental health, respiratory, surgical history, renal)

Staff name:	Designation:
Date:	Signature:

Patient NHS No:

MEDICATION

Allergies:		Reaction:	Treatment:
Diabetes medication	NAME	DOSE	FURTHER INFO
All other medication (inc. check for any cardiac or respiratory meds)	NAME	DOSE	FURTHER INFO

Staff name:	Designation:
Date:	Signature:

Patient NHS No:	

**Current level of physical activity**	Does the participant perform planned, structured physical activity of at least 30 min at moderate intensity on at least 3 days/week for at least the last 3 months?<br>Yes ☐ No ☐<br>What activities, if any, does the participant do during the week?<br>How far can the participant walk before needing to stop?<br>Less than a minute ☐ 2-5 minutes ☐ more than 5 minutes ☐<br>What stops them from walking further?<br>How much time do they spend sitting during a week day?<br>Less than an hour ☐ 1-3 hours ☐ More than 3 hours ☐<br>How much time do they spend sitting at the weekend?<br>Less than an hour ☐ 1-3 hours ☐ More than 3 hours ☐
**Mobility**	**Walking Aid**<br>Stick ☐ Scooter ☐ Frame/Mobilator ☐ Offloading footwear ☐<br>Other ☐
**Falls in last the year**	Yes ☐ No ☐<br>Falls history (number/circumstances):
	Any further details/issues:<br>Referral to falls clinic required?

Staff name:	Designation:
Date:	Signature:

Patient NHS No:

**Light intensity activity**<br>Light intensity activity requires minimal effort, whilst able to maintain a conversation and sing with ease. In the context of walking, light intensity is equivalent to under 100 steps per minute.
**Moderate intensity activity**<br>Moderate intensity activity involves working hard enough to raise the heart rate and break a sweat, whilst able to maintain a conversation but not sing. In the context of walking, moderate intensity is equivalent to between 100 and <130 steps per minute. Generally safe, although caution may be required in a minority of individuals with advanced forms of cardiovascular disease.

ECG/Medical history review	SIGN	DATE	NAME & DESIGNATION
From this participants medical history and ECG they are safe to exercise at a LIGHT INTENSITY (including breaking up sitting time)<br>Yes ☐ No ☐			
From this participants medical history and ECG they are safe to exercise at a MODERATE INTENSITY<br>Yes ☐ No ☐			
ECG notes:			

Staff name:	Designation:
Date:	Signature:

Patient NHS No:

FOR COMPLETION DURING INDIVIDUALISED ASSESSMENT:

HADS Questionnaire

**HADS – Anxiety**	**Score:**	**Notes:**
**HADS – Depression**	**Score:**	**Notes:**

**Date**		**Resting Heart Rate**	
**Sitting BP**		**Resting SpO2(%)**	
**Standing BP**		**Blood glucose**	

INDIVIDUALISED APPOINTMENT OUTCOME:

Medication started:

Medication stopped/ altered:

Are hypos an issue? Yes ☐ No ☐

Gold score (more than 4 indicates impaired hypo awareness):

Using CGM? Yes ☐ No ☐

Advised to check CBG or CGM device prior to activity? Yes ☐ No ☐
Comments:

Active foot ulcer? Yes ☐ No ☐
Is the foot ulcer on a weight bearing surface? Yes ☐ No ☐ details:

Any cast / boot / equipment / advice to follow (please give details):

Recommended weight bearing status

☐ Non weight bearing (e.g. due to active foot ulcer, chair based activity only)
☐ Partial weight bearing
☐ Full weight bearing (e.g. no active foot ulcer, OK to do standing activity / usual activities of daily living

Staff name:	Designation:
Date:	Signature:

Patient NHS No:

INDIVIDUALISED ASSESSMENT CHECKLIST

	Y/N	Comments (if needed)<br>(Document if not applicable)
Health profile completed with participant?		
Optimisation of results – HbA1c		
Optimisation of results – BP		
Optimisation of results – cholesterol		
Optimisation of results – eGFR		
Optimisation of results - ACR		
Review of HADS questionnaire		
Weight bearing status Documented		
Date offered for first group SME / physical activity session		

SUMMARY OF EXERCISE CONSIDERATIONS AND NEEDS

Is this participant safe to exercise? (please tick)

- ☐ **Yes – Moderate intensity**
(activity involves working hard enough to raise the heart rate and break a sweat, whilst able to maintain a conversation but not sing. In the context of walking, moderate intensity is equivalent to between 100 and <130 steps per minute. Generally safe, although caution may be required in a minority of individuals with advanced forms of cardiovascular disease)
- ☐ **Yes – Light intensity**
(activity requires minimal effort, whilst able to maintain a conversation and sing with ease. In the context of walking, light intensity is equivalent to under 100 steps per minute)
- ☐ **No – Would benefit from reducing sedentary behaviour only**
(e.g. due to contraindication or co-morbidity)
- ☐ **No – Requires further assessment or discussion**
(e.g. due to contraindication, concern or consideration due to co-morbidities, complex medical history or new or not yet investigated shortness of breath, chest pain/ tightness, dizziness or palpitations)

If no please give details:	(include onward referrals for medical clearance, and date sent)

Staff name:	Designation:
Date:	Signature:

Patient NHS No:

	**Individualised appointment assessor notes**	**MiFoot Physical activity reviewer notes**
**Suitable for Mifoot PA sessions?** (inc. any medical clearance detail)		
**Any precautions or adaptations to MiFoot PA sessions needed?**		
**Signature:**		
**Print name:**		
**Designation:**		
**Date:**		

MIFOOT PHYSICAL ACTIVITY SESSIONS:

	**Notes:** (Inc. any changes to health status or symptoms, any new concerns, HR/Sao2/blood glucose results from session, any onward referrals)	**Signature and date:**
WEEK 1<br>30 mins		
WEEK 2<br>45 mins		
WEEK 3<br>60 mins		
WEEK 4<br>60 mins		
WEEK 5<br>60 mins		
WEEK 6<br>60 mins		
WEEK 7<br>60 mins		

Staff name:	Designation:
Date:	Signature:

Patient NHS No:

BOOSTER SESSIONS

	**Notes:** (Inc. any changes to health status or symptoms, any new concerns, HR/Sao2/blood glucose results from session, any onward referrals)	**Signature and date:**
MONTH 1		
MONTH 2		
MONTH 3		
MONTH 4		
MONTH 5		
MONTH 6		
MONTH 7		
MONTH 8		
MONTH 18-24		

Staff name:	Designation:
Date:	Signature:

Patient NHS No:

CONTINUATION SHEET

Date	Notes:	Signature and date:

Staff name:	Designation:
Date:	Signature:
