# Supplementary material for: Expert considerations to guide the development and delivery of an exercise intervention in people with type 2 diabetes‐related foot ulcer disease
Source: Diabet Med. 2026 Apr 30;43(8):e70338. doi: 10.1111/dme.70338 (PMC13380388; doi:10.1111/dme.70338)
Supplement: Supplementary file 2 — Appendix S2. MiFoot physical activity monitoring sheet. [file DME-43-e70338-s002.pdf]

PHYSICAL ACTIVITY MONITORING - HEART RATE (HR) AND EXERTION

Name:	
HRrest:	HRmax:
Max exercise HR:	Exercise intensity: Light / moderate
Weight bearing status: Chair based only / partial / full (standing)	

	HRrest – start of session	Peak RPE – mid session	HR – mid session	HR – post session	Comments
Date:					
Date:					
Date:					
Date:					
Date:					
Date:					
Date:					

MiFoot
